# Repetitive Editing and RNA Splicing

**DOI:** 10.1371/journal.pbio.0020426

**Published:** 2004-11-09

The so-called “central dogma” of biology—DNA makes RNA makes protein—is a simple statement that subsumes a wealth of complexity. In particular, the past decade has shown that after RNA is made, it is run through a gauntlet of processes that strip it of introns and splice its exons, add a cap and tail, and even chemically modify one or more bases along the way. This last possibility includes deamination—removal of an NH_2_ group—from adenosine, converting it to inosine. During translation, the ribosome reads an inosine as a guanosine; thus, an A-to-I edit in RNA can even cause an amino acid change in the resulting protein. A new study in this issue by Stefan Maas and colleagues shows that A-to-I editing is remarkably widespread among human genes, and commonly targets a ubiquitous repetitive sequence, the Alu repeat.

A-to-I editing has been recognized for several years, but the known targets have been few, far fewer than the number predicted by measuring the inosine content in messenger RNA. To identify more targets, Alekos Athanasiadis, Alexander Rich, and Maas compared genomic sequences to cDNA sequences. Adenosines are unchanged in genomic DNA, while in complementary DNA (cDNA), which is derived from reverse transcribing mRNA, any adenosines that were converted to inosines during RNA editing show up as guanosines. Thus, A-to-G discrepancies revealed candidate editing sites. To reduce the number of false positives, the researchers confined their search to regions with multiple A-to-G discrepancies. In an initial screen of 3,000 cDNAs, they found 26 A-to-I edited genes. In all but one case, the editing occurred in an Alu sequence.[Fig pbio-0020426-g001]

**Figure pbio-0020426-g001:**
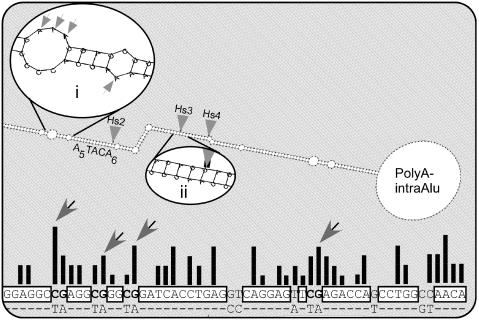
Sequence and structure preferences of editing in Alu repeats

There are approximately 1.4 million Alu sequences in the human genome, each about 300 base-pairs in length, which together comprise about 10% of the entire genome. Not all of them occur in genes, but those that do are typically found in transcribed but untranslated regions (introns), either upstream (3′) or downstream (5′) of the translated region. In many genes, they are found in pairs, ordered head to head or tail to tail, separated by a short intervening sequence. Once transcribed, the Alu sequences can pair up, forming a stretch of double-stranded RNA that makes an ideal target for the A-to-I RNA editing machinery, called ADAR (adenosine deaminase acting on RNA).

A typical gene contains between one and two dozen Alu sequences. Based on this and the frequency of editing found when analyzing more than 100,000 mRNAs in the human transcriptome, Athanasiadis, Rich, and Maas estimate that the probability that any particular mRNA undergoes A-to-I editing is between 85% and 95%.

While the bulk of edited Alu sites are in introns, a small fraction of them are in exons. Here they can lead to alternative forms of the same protein, expressed in different cell types or at different times; this appears to be especially common in the nervous system. Alu editing can also convert introns to exons, and vice versa, through creation or destruction of splice sites. It is possible A-to-I editing may be used to reduce the creation of deleterious new exons, although more work will be needed to explore this possibility, as well as what role, if any, A-to-I editing plays in promoting new exon creation.

